# Supplementary material for: Immune Responses to HBV Vaccine in People Living with HIV (PLWHs) Who Achieved Successful Treatment: A Prospective Cohort Study
Source: Vaccines (Basel). 2023 Feb 9;11(2):400. doi: 10.3390/vaccines11020400 (PMC9967144; doi:10.3390/vaccines11020400)
Supplement: Supplementary file 1 [file vaccines-11-00400-s001.zip › vaccines-2126418-supplementary.pdf]

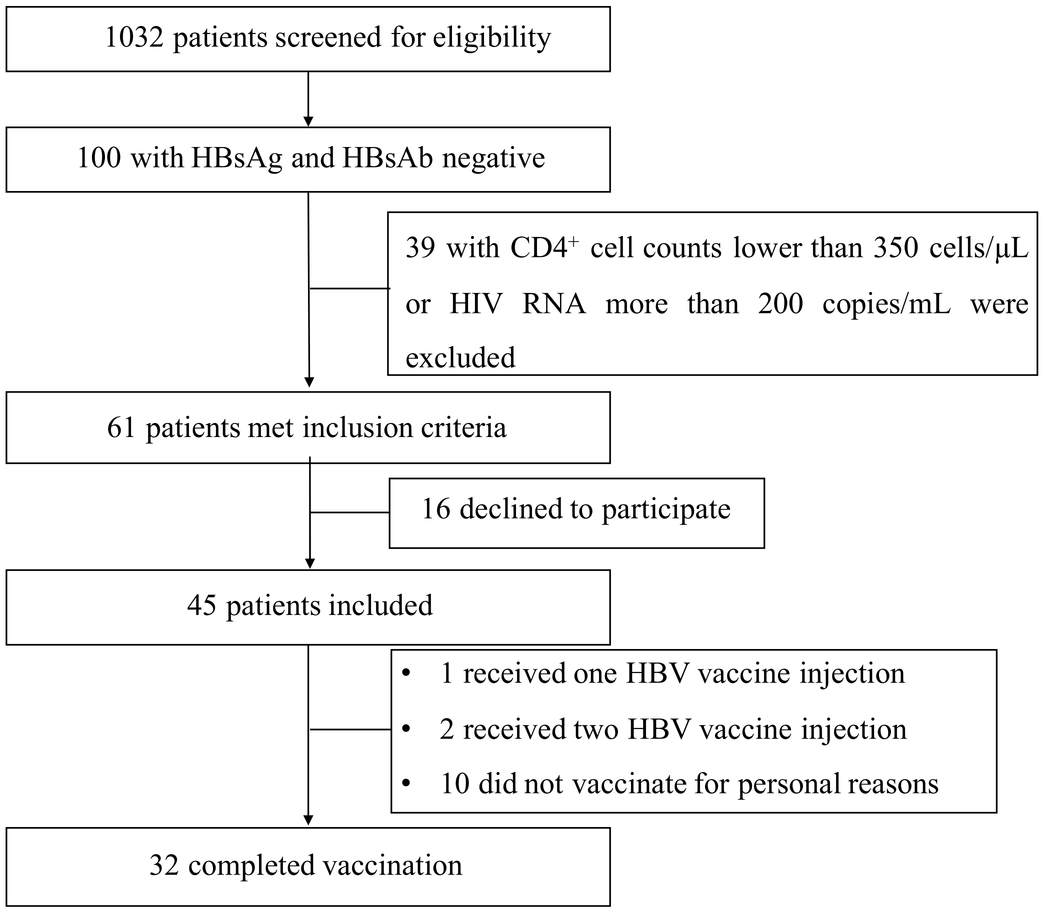

Figure S1. Flow chart for recruitment of the patients attended during the study period.

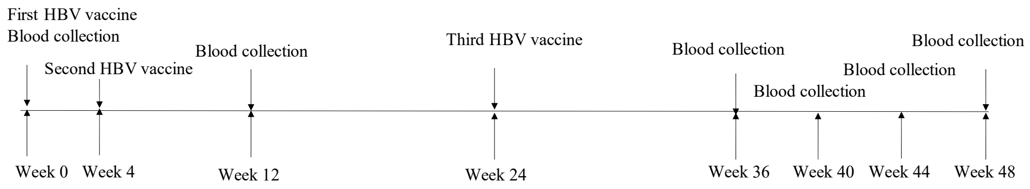

Figure S2. Timeline of vaccination and blood collection

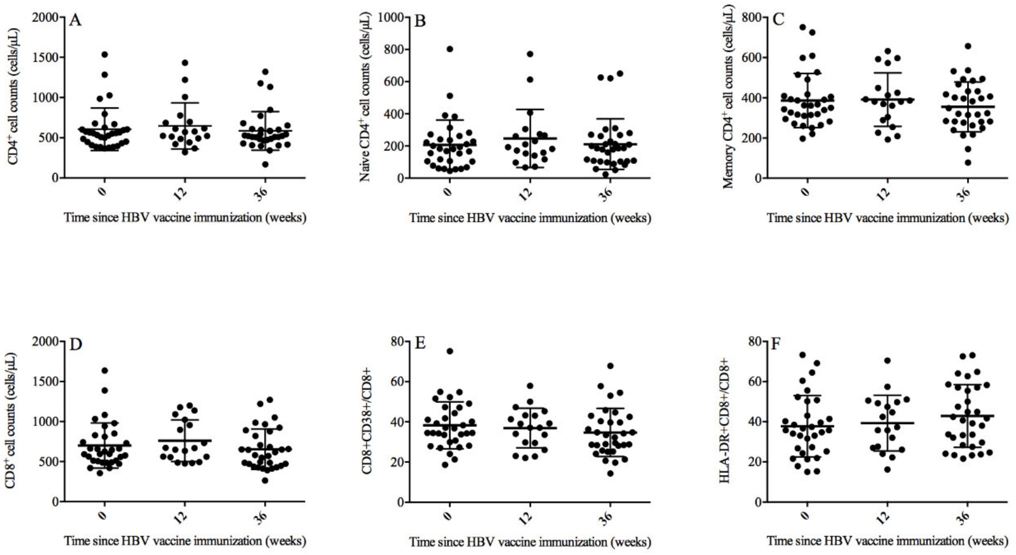

Figure S3. The changes in T cell subsets counts. The dynamics of $CD4^+$ cell count (A), naïve $CD4^+$ cell count (B), memory $CD4^+$ cell count (C), $CD8^+$ cell count (D), $CD38^+$ $CD8^+$ percentage(E), HLA-DR^+CD8^+ percentage (F) before HBV vaccine immunization, 12 weeks and 36 weeks after receiving the first HBV vaccine.

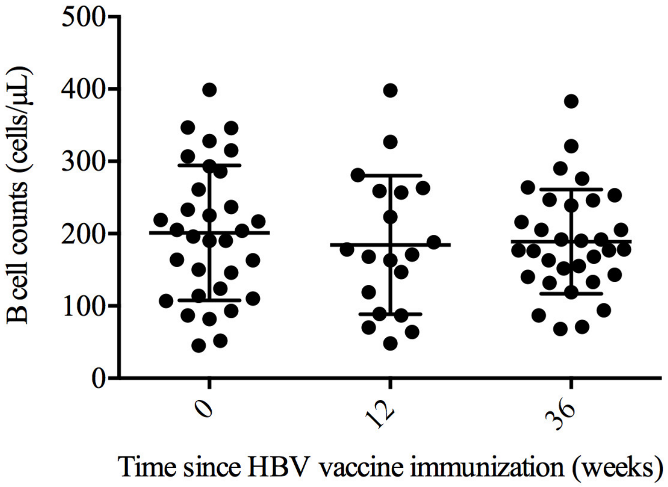

Figure S4. The changes in B cell counts during the follow-up period. There were no relevant alterations in B cell counts.
